# Correlation between the triglyceride-glucose index and left ventricular global longitudinal strain in patients with chronic heart failure: a cross-sectional study

**DOI:** 10.1186/s12933-024-02259-2

**Published:** 2024-05-29

**Authors:** Shuai Zhang, Yan Liu, Fangfang Liu, Qing Ye, Dachuan Guo, Panpan Xu, Tianhao Wei, Cheng Zhang, Huixia Lu

**Affiliations:** 1 State Key Laboratory for Innovation and Transformation of Luobing Theory, Jinan, China; 2 Key Laboratory of Cardiovascular Remodeling and Function Research, Chinese Ministry of Education, Chinese National Health Commission and Chinese Academy of Medical Sciences, Jinan, China; 3 Department of Cardiology, Qilu Hospital of Shandong University, Jinan, China

**Keywords:** Chronic heart failure, Triglyceride-glucose index, Global longitudinal strain

## Abstract

**Background:**

Left ventricular global longitudinal strain (GLS) holds greater diagnostic and prognostic value than left ventricular ejection fraction (LVEF) in the heart failure (HF) patients. The triglyceride-glucose (TyG) index serves as a reliable surrogate for insulin resistance (IR) and is strongly associated with several adverse cardiovascular events. However, there remains a research gap concerning the correlation between the TyG index and GLS among patients with chronic heart failure (CHF).

**Method:**

427 CHF patients were included in the final analysis. Patient demographic information, along with laboratory tests such as blood glucose, lipids profiles, and echocardiographic data were collected. The TyG index was calculated as Ln [fasting triglyceride (TG) (mg/dL) × fasting plasma glucose (FPG) (mg/dL)/2].

**Results:**

Among CHF patients, GLS was notably lower in the higher TyG index group compared to the lower TyG index group. Following adjustment for confounding factors, GLS demonstrated gradual decrease with increasing TyG index, regardless of the LVEF level and CHF classification.

**Conclusion:**

Elevated TyG index may be independently associated with more severe clinical left ventricular dysfunction in patients with CHF.

**Supplementary Information:**

The online version contains supplementary material available at 10.1186/s12933-024-02259-2.

## Introduction

Heart failure (HF) is a major global medical challenge with an increasing prevalence and poor prognosis [1, 2]. Traditionally, HF has been broadly categorized according to the left ventricular ejection fraction (LVEF) into three groups: heart failure with reduced ejection fraction (HFrEF, LVEF is ≤ 40%), heart failure with mildly-reduced ejection fraction (HFmrEF, LVEF 41–49%), and heart failure with preserved ejection fraction (HFpEF, LVEF ≥ 50%), each exhibiting varying degrees of systolic and diastolic dysfunction [3, 4]. While LVEF, typically assessed via echocardiography, remains pivotal for HF diagnosis, characterization, prognosis, patient triage, and treatment selection [5, 6], However, this parameter is constrained not only by technical limitations but also by pathophysiological factors, including situations where the ratio of stroke volume to left ventricular (LV) cavity size remains unchanged [7]. Moreover, LVEF fails to differentiate between healthy hearts and HFpEF patients and inadequately reflects actual cardiac function [8, 9]. Strain analysis, emerging as a promising tool for evaluating cardiac contractility and myocardial deformation, offers a more comprehensive characterization of patients [10]. A growing number of studies have demonstrated that strain provides better predictor of prognostic value in HF patients compared to LVEF [5, 11–13].

Insulin resistance (IR), indictive of metabolic disorders and systemic inflammation, is an independent and significant risk factor for HF [14–16]. However, the gold standard method for measuring IR, the hyperinsulinemic-euglycemic clamp (HIEC), is time-consuming and invasive [17, 18], limiting its clinical applicability. The triglyceride-glucose (TyG) index has recently been regarded as a simpler, cost-effective, and reliable surrogate marker of IR, demonstrating high concordance with the HIEC [19–21]. Previous studies have found that the TyG index may play an essential role in the impairment of left ventricular structure and function [22, 23] and is associated with the development of HF and poor prognosis [24–27]. Na et al. reported an independent association between the higher TyG index and reduced GLS in patients with coronary artery disease (CAD) [28].

Although several recent studies have established a link between the TyG index and left ventricular function, no study has explored the relationship between the TyG index and GLS in CHF. Thus, we investigated the relationship between the TyG index and GLS in CHF patients and, for the first time, examining its relevance across different HF groups.

## Methods

### Study population

The study adhered to the principles outlined in the Declaration of Helsinki and was approved by the Ethics Review Committee of Qilu Hospital of Shandong University.

**Fig. 1:**
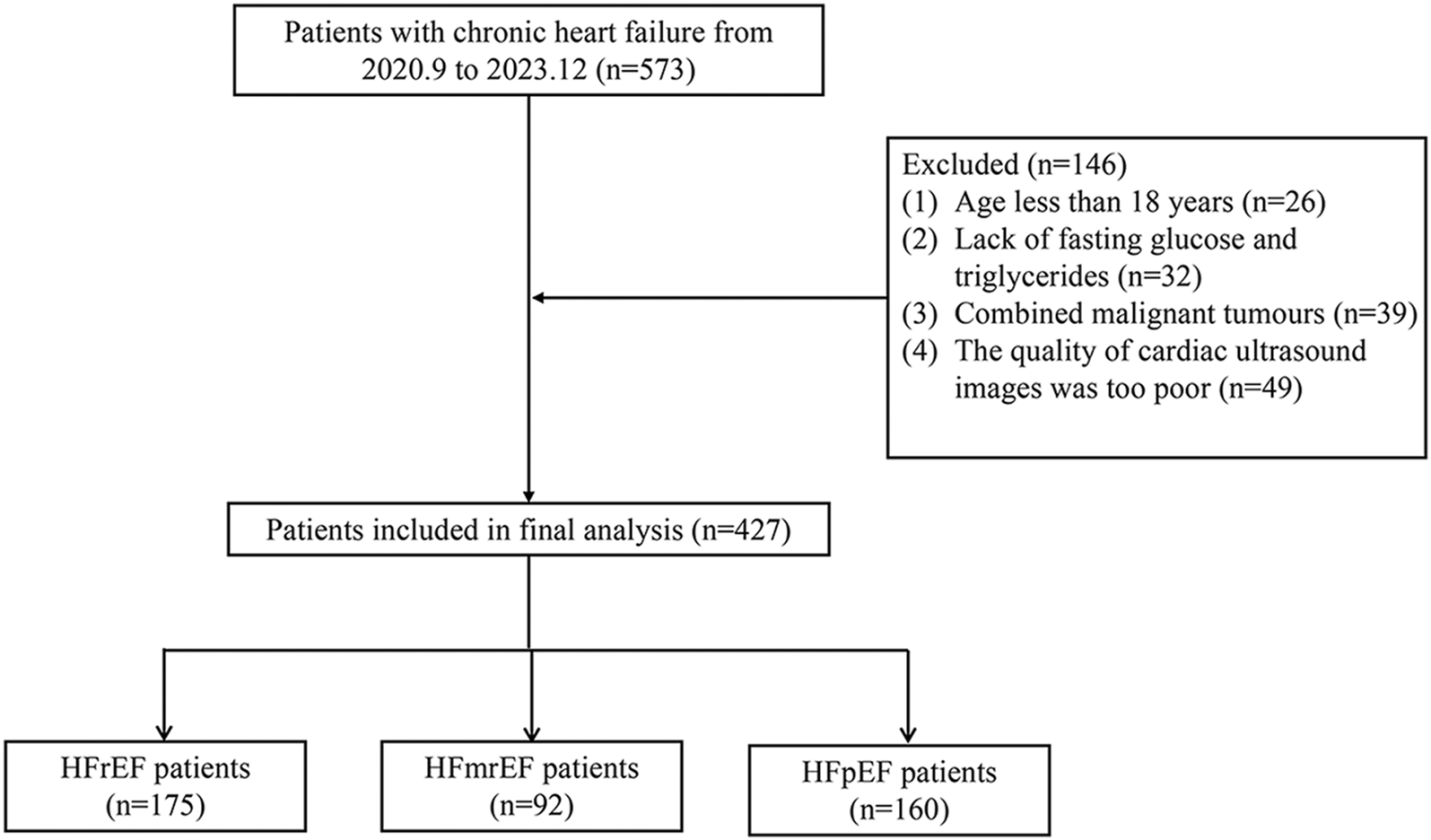
Flow diagram of patient selection

This retrospective study spanned from September 2020 to December 2023 and involved 573 consecutive patients presenting with CHF at Qilu Hospital of Shandong University. Among them, 49 patients were excluded due to poor image quality, 32 patients lacked fasting glucose and triglycerides, 26 patients were younger than 18 years old, and 39 patients had malignant neoplasms. Ultimately, 427 patients with CHF were included in the final analysis, comprising 175 HFrEF, 92 HFmrEF, and 160 HFpEF (Fig. 1).

### Echocardiographic measurement

Echocardiographic images were acquired by an experienced echocardiogram physician. Adhering to the 2016 guidelines provided by the American Society of Echocardiography and the European Society of Cardiovascular Imaging, participants were applied to the anterior chest area and connected to a three-lead ECG and we instructed all participants to hold their breath during image acquisition to ensure high-quality images [29]. The left ventricular end systolic volume (LVESV), left ventricular end diastolic volume (LVEDV), left ventricular end diastolic dimension (LVDd), left ventricular end systolic dimension (LVDs), left ventricular posterior wall thickness (LVPWT), interventricular septal thickness (IVST), LVEF and GLS was acquired by GEVividE95 color ultrasound diagnostic instrument (GE of USA) with an M5s probe and a frequency $1.5 \sim 3.6$ MHz.

### Data collection and definitions

Data on demographic characteristics, medical history, personal history, and medication usage were collected through an electronic medical record system. Venous blood samples were drawn after overnight fasting to measure levels of N-terminal pro-brain natriuretic peptide (NT-proBNP), fasting plasma glucose (FPG), serum creatinine (SCr), and lipid profile. CHF was defined according to the 2021 ESC Guidelines for the diagnosis and treatment of acute and chronic heart failure [30]. Body mass index (BMI) was calculated as weight (kg) divided by the square of height (m^2^). Hypertension was defined as systolic blood pressure ≥ 140 mmHg and/or diastolic blood pressure ≥ 90 mmHg, or the use of antihypertensive medication. Diabetes mellitus (DM) was defined as an FPG level ≥ 7.0 mmol/L or 2 h plasma glucose level ≥ 11.1 mmol/L following an oral glucose tolerance test (OGTT), or the use of oral hypoglycemic agents or insulin [31]. Hyperlipidemia was diagnosed with and ICD-10 code E78 along with lipid-lowering medication, or a total serum cholesterol ≥ 240 mg/dL) [32]. CAD was confirmed by the presence of at least one major coronary artery with ≥ 50% stenosis as evaluated by coronary angiography (CAG), including the left anterior descending, left circumflex, and right coronary arteries [33]. The diagnosis of obstructive sleep apnea (OSA) relied on an AHI ≥ 5/h, accompanied by suggestive symptoms according to the International Classification of Sleep Disorders (Third Edition) criteria [34]. Patients with a self-reported diagnosis of hypertension, DM, hyperlipidemia, CAD, or OSA, substantiated by corresponding medical records, were also identified as having these conditions. The estimated glomerular filtration rate (eGFR) was calculated using the following equations: eGFR (mL/min/1.73 m^2^) = 175 × SCr (mg/dL)  ^−1.234^ × age (year) ^−0.179^ × 0.79 (in the case of women) [35]. Chronic kidney disease (CKD) was defined as an eGFR < 60 mL/min/1.73 m^2^. The TyG index was determined by the formula: Ln [fasting triglyceride (TG) (mg/dL) × FPG (mg/dL)/2] [21]. Reduced GLS is now defined as GLS < 11.2%, which represents the value of GLS less than the median in HF. Left ventricular mass (LVM, g) = 0.8 [1.04 (LVDd + LVPWT + LVST)^3^− (LVDd)^3^] + 0.6. Body surface area (BSA, m^2^) = 0.0061*height (cm) + 0.0128*weight (kg) − 0.1529. Left ventricular mass index (LVMi, g/m^2^) = LVM (g)/BAS(m^2^).

## Statistical analysis

Statistical analysis was performed using SPSS version 25.0 (SPSS, Chicago, IL, United States), R software version 4.2.0 (R Foundation for Statistical Computing, Vienna, Austria), and GraphPad PRISM version 10.0 (GraphPad Software—San Diego, CA, USA). Initially, we assessed the baseline characteristics of the overall enrolled population and categorized them according to the tertile of the TyG index. Continuous variables were presented as mean ± standard deviation (SD) or median (interquartile range) and comparisons were made using Student’s t-test or Mann–Whitney U-test as appropriate. Categorical variables were expressed as counts and percentages and analyzed using the chi-square or Fisher exact tests. We used Pearson or Spearman correlation analysis to evaluate the association between the TyG index and cardiovascular risk factors in CHF, HFrEF, HFmrEF, and HFpEF. To mitigated bias from multicollinearity, we calculated the variance inflation factor (VIF) of the variables in the model (Additional file 1: Table S1). We did not find evidence of collinearity in the models, given the VIF of < 10. The association between the TyG index and GLS in CHF and its three subtypes was assessed using multivariable linear regression. Three models were constructed following adjusting for potential confounders: model 1 adjusted for age and gender; model 2 further adjusted for BMI, hypertension, CAD, DM, hyperlipidemia, OSA, smoking, and drinking; and model 3 additionally adjusted for LVEF, NT-proBNP, total cholesterol (TC), low-density lipoprotein cholesterol (LDL-C), high-density lipoprotein cholesterol (HDL-C), eGFR, E/e’, and LVMi. Furthermore, the TyG index was progressively included as both a continuous and categorical variable in multivariable logistic regression. To further explore the relationship between the TyG index and GLS, we employed restricted cubic spline curve (RCS) analysis. Subgroup analysis was conducted based on gender, hypertension, hyperlipidemia, DM, CAD, and BMI to assess potential variations in the association between the TyG index and GLS, with interaction P-values calculated accordingly. Finally, sensitivity analysis was performed by excluding patients with a history of lipid-lowering or glucose-lowering drug use, a history of sodium-glucose cotransporter-2 inhibitors (SGLT-2i) usage, and those with DM. Statistical significance was set at P-values less than 0.05.

## Results

427 CHF patients were included in this study with a mean age of 48.37 ± 14.50 years, and 271 (63.5%) were males. As shown in Table 1, the patients were stratified into 3 groups according to the tertiles of the TyG index (tertile 1: n = 143, TyG index < 8.38; tertile 2: n = 141, 8.38 ≤ TyG index < 8.82; and tertile 3: n = 143, TyG index ≥ 8.82). As illustrated in Table 1, higher baseline TyG index was associated with increased prevalence of DM and hyperlipidemia as well as lower LVEF and GLS, and a higher ratio of males and patients with a history of coronary artery bypass grafting (CABG). Statistical significance was also found in other parameters such as E/e’, FPG, TC, LDL-C, HDL-C, TG, and hypoglycemic drug usage (all P-values < 0.05). No significant difference was observed in the other indicators (Table 1). Further comparisons of baseline characteristics of HFrEF, HFmrEF, and HFpEF was showed in Additional file 1: Table S2.

**Table 1:** Baseline characteristics of the study population according to the tertile of the TyG index

Variables	Total (n = 427)	Tertile 1 (n = 143)	Tertile 2 (n = 141)	Tertile 3 (N = 143)	P-value
TyG index	8.63 ± 0.50	8.10 ± 0.19	8.59 ± 0.13	9.20 ± 0.33	** < 0.001**
General conditions					
Age (years)	48.37 ± 14.50	49.43 ± 15.73	47.43 ± 13.82	48.24 ± 13.89	0.526
Male, n (%)	271 (63.5)	82 (57.3)	82 (58.2)	107 (74.9)	**0.011**
BMI (kg/m^2^)	27.09 ± 6.87	26.55 ± 7.70	26.66 ± 6.30	28.06 ± 6.46	0.116
Smoking, n (%)	182 (43.3)	52 (36.9)	59 (42.8)	71 (50.4)	0.073
Drinking, n (%)	193 (46.0)	63 (44.7)	61 (44.2)	69 (48.9)	0.681
Medical history, n (%)					
Hypertension	156 (36.5)	46 (32.2)	47 (33.3)	63 (44.1)	0.071
DM	68 (16.2)	13 (9.2)	21 (15.4)	34 (23.9)	**0.003**
Hyperlipidemia	164 (38.4)	45 (31.5)	51 (36.2)	68 (47.6)	**0.016**
CAD	89 (21.1)	23 (16.2)	32 (23.2)	34 (24.1)	0.204
Pervious PCI	44 (10.5)	14 (9.9)	14 (10.1)	16 (11.3)	0.910
Pervious CABG	13 (3.1)	1 (0.7)	3 (2.2)	9 (6.4)	**0.017**
OSA	43 (10.2)	12 (8.5)	15 (10.9)	16 (11.2)	0.689
CKD	45 (10.5)	17 (11.9)	12 (8.5)	16 (11.2)	0.620
Echocardiographic					
LVEF (%)	44.02 ± 13.79	46.00 (36.00–54.00)	45.00 (36.00–54.00)	43.00 (30.00–54.00)	**0.048**
GLS (%)	11.48 ± 4.28	13.10 ± 4.18	11.69 ± 3.89	9.67 ± 4.08	** < 0.001**
LVEDV (ml)	137 (104–185)	130 (104–171)	138 (106–180)	142 (103–206)	0.214
LVSDV (ml)	65 (50–92)	65 (48–88)	65 (52–91)	64 (50–103)	0.357
LVDd (mm)	58.0 (53.0–66.0)	57.5 (53.0–65.0)	58.0 (53.0–67.0)	58.0 (51.3–66.0)	0.872
LVDs (mm)	42.0 (36.0–52.0)	41.0 (37.0–49.3)	43.0 (36.5–53.0)	42.0 (35.3–54.0)	0.612
E (cm/s)	73.72 ± 25.01	70.50 (53.25–85.75)	76.00 (57.00–93.25)	68.50 (54.00–86.00)	0.106
E/e’	10.68 (7.90–14.70)	9.70 (7.40–12.09)	11.20 (7.86–14.55)	11.21 (8.40–14.47)	**0.011**
LVM (g)	156.75 (98.91–233.09)	158.96 (91.76–229.51)	153.42 (91.76–230.97)	166.19 (112.10–251.30)	0.119
LVMi (g/m^2^)	88.00 (54.70–128.05)	89.19 (54.46–123.71)	83.72 (51.54–120.94)	88.03 (57.13–135.81)	0.377
Laboratory text					
FPG (mmol/L)	5.29 (4.91–5.84)	4.97 (4.65–5.23)	5.27 (5.00–5.67)	5.94 (5.37–7.09)	** < 0.001**
TC (mmol/L)	4.06 (3.42–4.92)	3.89 (3.18–4.51)	4.18 (3.42–4.92)	4.40 (3.57–5.18)	**0.001**
LDL-C (mmol/L)	2.46 ± 0.87	2.30 ± 0.77	2.44 ± 0.88	2.64 ± 0.91	**0.004**
HDL-C (mmol/L)	1.11 (0.97–1.36)	1.18 (1.01–1.50)	1.14 (0.98–1.40)	1.03 (0.91–1.20)	** < 0.001**
TG (mmol/L)	1.23 (0.97–1.64)	0.96 (0.74–1.08)	1.25 (1.14–1.40)	1.89 (1.56–2.46)	** < 0.001**
eGFR(ml/min/1.73m^2^)	94.12 ± 30.60	94.77 ± 32.43	95.33 ± 29.98	92.28 ± 29.43	0.497
NT-proBNP (pg/ml)	474 (166–1276)	467 (166–1597)	470 (199–1569)	489 (144–1080)	0.633
Cardiovascular medications, n (%)					
Hypoglycemic drugs	68 (15.9)	12 (8.4)	19 (13.5)	37 (25.9)	** < 0.001**
Statins	112 (26.2)	36 (25.2)	35 (24.8)	41 (28.7)	0.716
Beta-blockers	364 (85.2)	123 (86.0)	115 (81.6)	126 (88.1)	0.283
ACEI/ARB/ANRI	331 (77.5)	103 (72.0)	113 (80.1)	115 (80.4)	0.156
MRA	308 (72.1)	97 (67.8)	102 (72.3)	109 (76.2)	0.285
SGLT-2i	209 (48.9)	67 (46.9)	73 (51.8)	69 (48.3)	0.694
HF group, n (%)					0.489
HFrEF	175 (41.0)	53 (37.1)	56 (39.7)	66 (46.2)	
HFmrEF	92 (21.5)	30 (21.0)	31 (22.0)	31 (21.7)	
HFpEF	160 (37.5)	60 (42.0)	54 (38.3)	46 (32.2)	
HF etiology, n (%)					0.883
Severe valvular heart disease	31 (7.3)	12 (8.4)	7 (5.0)	12 (8.4)	
Hypertrophic cardiomyopathy	58 (13.6)	20 (14.0)	20 (14.2)	18 (12.6)	
Dilated cardiomyopathy	118 (27.6)	36 (25.2)	44 (31.2)	38 (26.6)	
Amyloidosis cardiomyopathy	19 (4.4)	5 (3.5)	5 (3.5)	9 (6.3)	
Ischemic heart disease	103 (24.1)	36 (25.2)	31 (22.0)	36 (25.2)	
Other causes	98 (23.0)	34 (23.8)	34 (24.1)	30 (21.0)	

*TyG* index triglyceride-glucose index, *BMI* body mass index, *DM* diabetes mellitus, *PCI* percutaneous coronary intervention; *CABG* coronary artery bypass graft surgery, *OSA* obstructive sleep apnea, *CKD* chronic kidney disease, *LVEF* left ventricular ejection fraction, *GLS* global longitudinal strain, *LVEDV* left ventricular end-diastolic volume, *LVESV* left ventricular end-systolic volume, *LVDd* left ventricular end diastolic dimension, *LVDs* left ventricular end systolic dimension, E early diastolic mitral valve peak E velocity, *E/e*’ the ratio of early diastolic mitral inflow velocity to septal mitral annulus tissue relaxation velocity in early diastole, *LVMi* left ventricular mass index, *FPG* fasting plasma glucose, *TC* total cholesterol, *LDL-C* low-density lipoprotein cholesterol, *HDL-C*, high-density lipoprotein cholesterol, *TG* triglyceride, *eGFR* estimated glomerular filtration rate, *NT-proBNP* N-terminal pro-brain natriuretic peptide; *ACEI* angiotensin converting enzyme inhibitor, *ARB* angiotensin receptor blocker, *ARNI* angiotensin receptor-neprilysin inhibitors, *SGLT-2i* sodium-glucose co-transporter-2 inhibitors, *HF* heart failure, *HFrEF* heart failure with reduced ejection fraction, *HFmrEF* heart failure with mildly-reduced ejection fraction, *HFpEF* heart failure with preserved ejection fraction

P values in bold are < 0.05

**Fig. 2:**
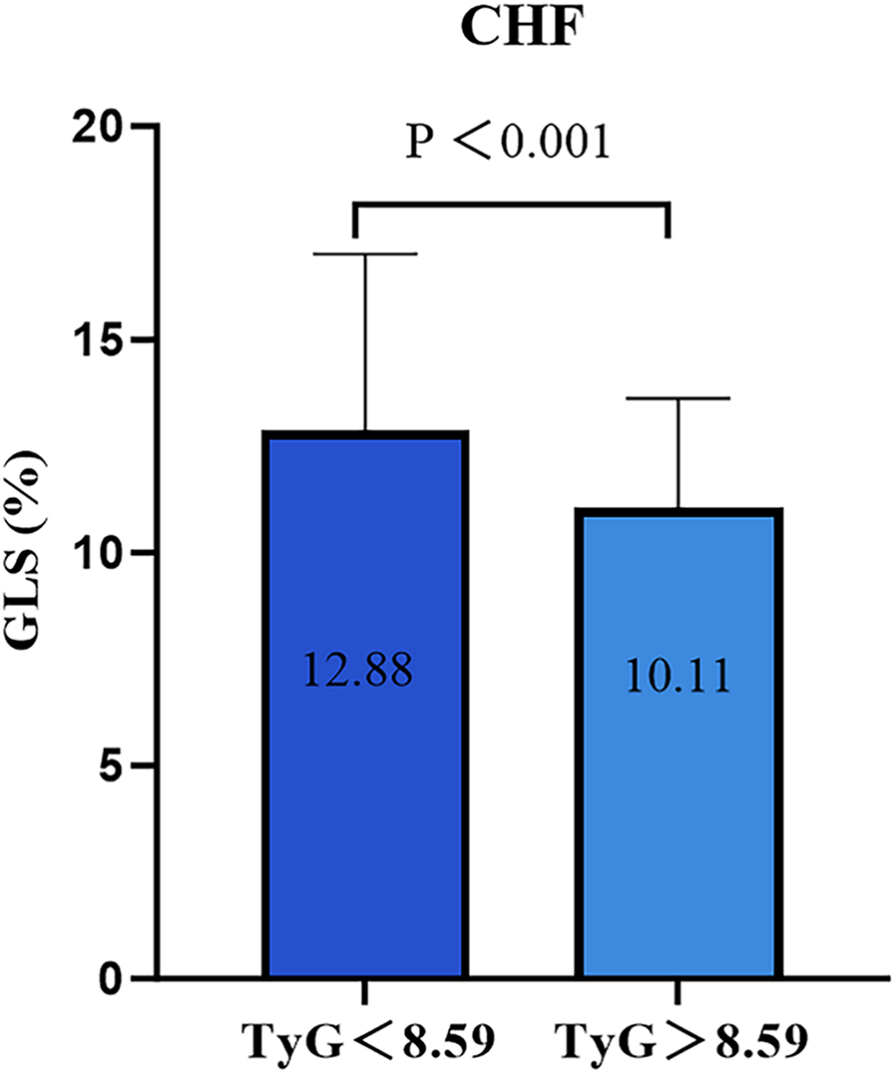
Comparison of GLS of high TyG index group with low TyG index. *TyG* index triglyceride-glucose index, *GLS* global longitudinal strain

**Fig. 3:**
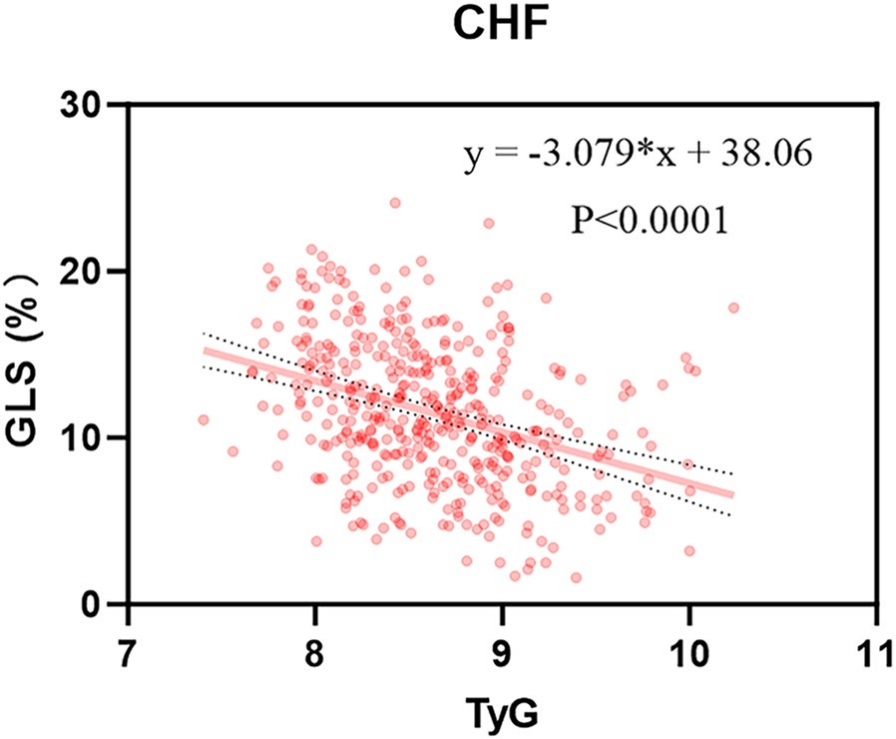
Scatterplot of GLS and TyG index for CHF. *TyG* index triglyceride-glucose index *GLS* global longitudinal strain, *CHF* chronic heart failure

In CHF patients, there was a significant reduction in GLS with increasing TyG index, as depicted in Fig. 2 and Fig. 3. We plotted histograms and scatter plots in HFrEF, HFmrEF, and HFpEF and obtained similar results in HFrEF, HFmrEF, and HFpEF patients (Additional file 1: Figures S1, S2). Pearson or Spearman correlation analysis revealed significant positive correlations between the TyG index and TC and LDL-C (all P-values < 0.05) and negative association with HDL-C, LVEF, and GLS (all P-values < 0.05) (Table 2), with GLS demonstrating the strongest negative correlation (r = − 0.365, P < 0.001). Additional correlation analysis for the three HF types (HFrEF, HFmrEF, and HFpEF) are detailed in Additional file 1: Table S3.

**Table 2:** Correlation between the TyG index and cardiovascular risk factors in CHF

Variables	Correlation coefficient (r)	P-value
Age(years)	− 0.035^a^	0.468
BMI(kg/m^2^)	0.073^a^	0.134
TC (mmol/L)	0.213^b^	** < 0.001**
LDL-C (mmol/L)	0.168^a^	**0.001**
HDL-C (mmol/L)	− 0.254^b^	** < 0.001**
eGFR (ml/min/1.73m^2^)	− 0.056^a^	0.252
NT-proBNP (pg/ml)	− 0.028^b^	0.567
LVEF (%)	− 0.133^a^	**0.006**
GLS (%)	− 0.365^a^	** < 0.001**

*TyG* index, triglyceride-glucose index, *CHF* chronic heart failure, BMI body mass index, *TC* total cholesterol, *LDL-C* low-density lipoprotein cholesterol, *HDL-C*, high-density lipoprotein cholesterol, *eGFR* estimated glomerular filtration rate, *NT-proBNP* N-terminal pro-brain natriuretic peptide, *LVEF* left ventricular ejection fraction, *GLS* global longitudinal strain

P-values in bold are < 0.05

^a^Person

^b^Spearman

Multivariable linear regression models were constructed to explore the independent association between the TyG index and GLS in CHF (Table 3). The β coefficients (95%CI) for the association between a 1-unit increase in the TyG index and decrease in GLS were documented as − 2.94 (− 3.70 to − 2.18), − 3.06 (− 3.84 to − 2.28), and − 1.99 (− 2.55 to − 1.44) in models 1, 2, and 3, respectively. In addition, when the TyG index was included as a categorical variable in the regression model, a significant linear trend with GLS was observed with a P-value < 0.05. In model 3, GLS for the highest tertile of TyG index decreased by 2.38 units [β (95% CI) − 2.38 (− 3.08 to − 1.68), P < 0.001], compared to the lowest tertile.

**Table 3:** Multivariable linear regression between the TyG index and GLS

TyG	β	95% CI	P-value
Model 1			
Per 1 unit	− 2.94	− 3.70 to − 2.18	< 0.001
Tertile 1	Ref.	Ref.	
Tertile 2	− 1.42	− 2.36 to − 0.48	0.003
Tertile 3	− 3.25	− 4.20 to − 2.30	< 0.001
Model 2			
Per 1 unit	− 3.06	− 3.84 to − 2.28	< 0.001
Tertile 1	Ref.	Ref.	
Tertile 2	− 1.54	− 2.50 to − 0.58	0.002
Tertile 3	− 3.44	− 4.42 to − 2.45	< 0.001
Model 3			
Per 1 unit	− 1.99	− 2.55 to − 1.44	< 0.001
Tertile 1	Ref.	Ref.	
Tertile 2	− 1.24	− 1.93 to − 0.55	< 0.001
Tertile 3	− 2.38	− 3.08 to − 1.68	< 0.001

Model 1: adjusted for age and gender

Model 2: adjusted for variables:model 1 covariates + BMI, hypertension, CAD, DM, hyperlipidemia, OSA, smoking, drinking

Model 3: adjusted for variables:model 2 covariates + LVEF, NT-proBNP, TC, LDL-C, HDL-C, eGFR, E/e’, and LVMi

*TyG* index triglyceride-glucose index, *GLS* global longitudinal strain, *Ref*. reference *BMI* body mass index, *CAD* coronary artery disease, *DM* diabetes mellitus, *OSA* obstructive sleep apnea, *LVEF* left ventricular ejection fraction, *NT*-proBNP N-terminal pro-brain natriuretic peptide, *TC* total cholesterol, *LDL-C* low-density lipoprotein cholesterol, *HDL-C* high-density lipoprotein cholesterol, *eGFR* estimated glomerular filtration rate, *E/e*’ the ratio of early diastolic mitral inflow velocity to septal mitral annulus tissue relaxation velocity in early diastole, *LVMi* left ventricular mass index

P values in bold are < 0.05

**Fig. 4:**
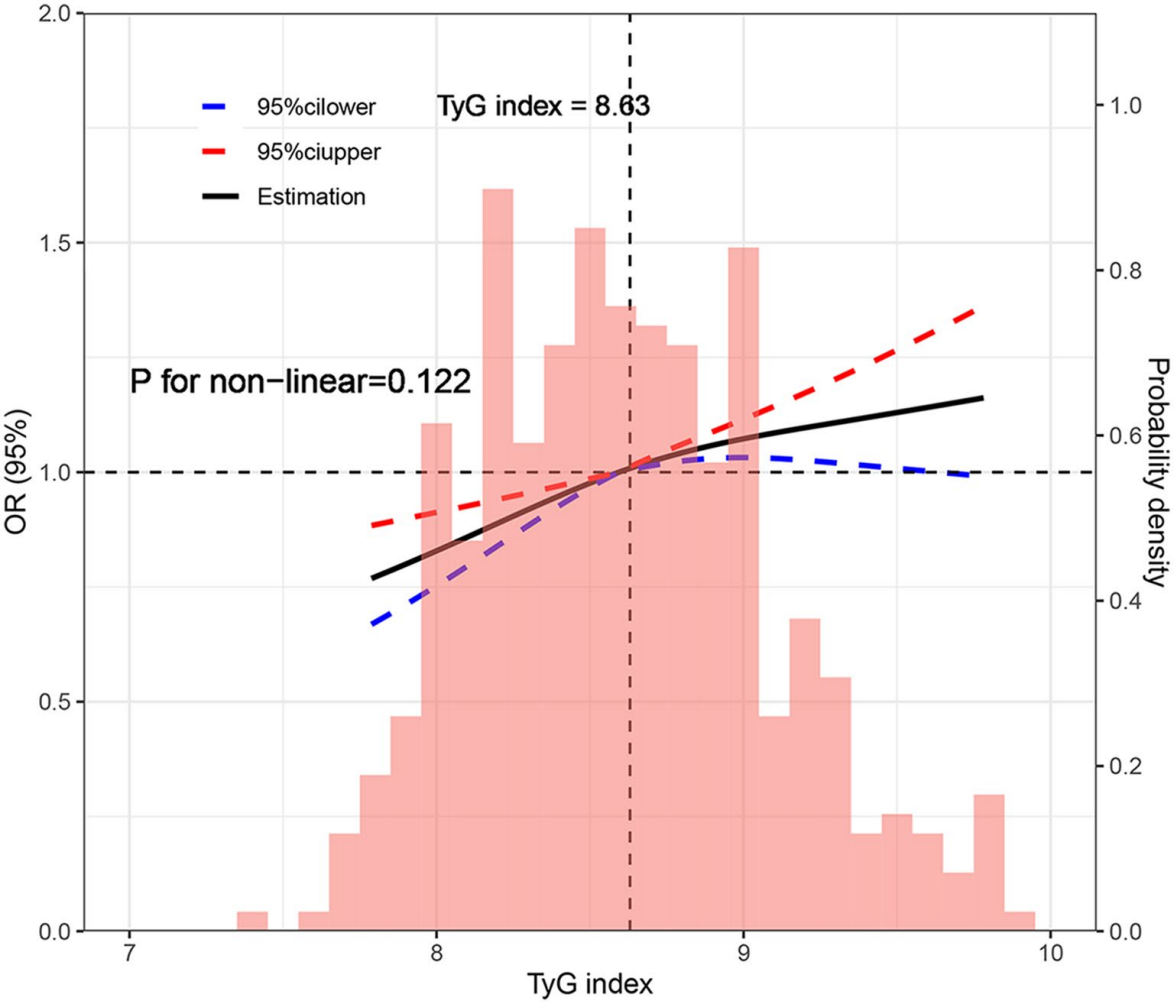
Restricted cubic spline plot between the TyG index and reduced GLS. Reduced GLS: GLS < 11.2%. Adjusted for age, gender, BMI, hypertension, CAD, DM, hyperlipidemia, OSA, smoking, drinking, LVEF, NT-proBNP, TC, LDL-C, HDL-C, eGFR, E/e’, LVMi. TyG index triglyceride-glucose index, *GLS* global longitudinal strain *BMI* body mass index, *CAD* coronary artery disease, *DM* diabetes mellitus, *OSA* obstructive sleep apnea, *LVEF* left ventricular ejection fraction, *NT-proBNP* N-terminal pro-brain natriuretic peptide, *TC* total cholesterol, *LDL-C* low-density lipoprotein cholesterol, *HDL-C*, high-density lipoprotein cholesterol, *eGFR* estimated glomerular filtration rate, *E/e*’ the ratio of early diastolic mitral inflow velocity to septal mitral annulus tissue relaxation velocity in early diastole, *LVMi* left ventricular mass index

Multivariable logistic regression analysis revealed the TyG index as a risk factor for reduced GLS (Table 4). The risk of reduced GLS in CHF patients increased by 244%, 299%, and 337% with a 1-unit increase in the TyG index in models 1, 2, and 3, respectively. Similarly, compared to tertile 1, the risk of reduced GLS in tertile 3 surged by 306%, 398%, and 549% in models 1, 2, and 3, respectively. Similarly, we utilized multivariable linear and logistic regression analysis to investigated whether the TyG index is independently correlated with GLS in HFrEF, HFmrEF, and HFmrEF, with results being provided in Additional file 1: Tables S4, S5. We evaluated the continuous relationship between the TyG index and GLS using RCS based on a multivariable logistic regression model (Fig. 4). Following adjustments for traditional factors, the TyG index demonstrated a gradual increase, accompanied by a corresponding rise in the OR of the lower GLS. Furthermore, we explored the relationship between the TyG index and GLS in the three types of HF using RCS (Additional file 1: Figure S3). To validate the relationship between the TyG index and GLS, we conducted additional sensitivity analyses. Specifically, we excluded three groups of individuals from the analysis: group 1: those with DM; group 2: those with a history of lipid-lowering or hypoglycemic usage; group 3: those with a history of SGLT-2i usage (Table 5). The correlation between the TyG index and GLS was also present in three groups, which may indicate that the correlation is independent of medication and DM. We performed corresponding sensitivity analyses in different HF types (Additional file 1: Table S6).

**Table 4:** Association between the TyG index and reduced GLS in different logistic models

TyG	OR (95% CI)		
	Model 1	Model 2	Model 3
Per 1 unit increase	3.44 (2.22–5.32)***	3.99 (2.50–6.37) ***	4.37 (2.37–8.03)**
Tertile 1	Ref.	Ref.	Ref.
Tertile 2	1.73 (1.07–2.80)*	1.91 (1.16–3.16)*	2.18 (1.09–4.34)*
Tertile 3	4.06 (2.46–6.70)***	4.98 (2.89–8.56)***	6.49 (3.03–11.86)***
P for trend	** < 0.001**	** < 0.001**	** < 0.001**

Reduced GLS: GLS < 11.2%

Model 1: adjusted for age and gender

Model 2: adjusted for variables:model 1 covariates + BMI, hypertension, CAD, DM, hyperlipidemia, OSA, smoking, drinking

Model 3: adjusted for variables:model 2 covariates + LVEF, NT-proBNP, TC, LDL-C, HDL-C, eGFR, E/e’, and LVMi

TyG index triglyceride-glucose index, GLS global longitudinal strain, Ref. reference BMI body mass index, CAD coronary artery disease, DM diabetes mellitus, OSA obstructive sleep apnea, LVEF left ventricular ejection fraction, NT-proBNP N-terminal pro-brain natriuretic peptide, TC total cholesterol, LDL-C low-density lipoprotein cholesterol, HDL-C, high-density lipoprotein cholesterol, eGFR estimated glomerular filtration rate, E/e’ the ratio of early diastolic mitral inflow velocity to septal mitral annulus tissue relaxation velocity in early diastole, LVMi left ventricular mass index

P values in bold are < 0.05

*P < 0.05, **P < 0.01, ***P < 0.001

**Table 5:** Sensitivity analysis for the association between the TyG index and GLS

	β	95% CI	P-value
Group 1	− 2.12	− 2.72 to − 1.51	< 0.001
Group 2	− 5.34	− 6.89 to − 3.80	< 0.001
Group 3	− 1.82	− 2.70 to − 0.94	< 0.001

Group 1:patients without DM

Group 2: patients without hypoglycemic or lipid-lowering drug usage

Group 3: patients without SGLT-2i usage

Adjusted for age, gender, BMI, hypertension, CAD, hyperlipidemia, OSA, smoking, drinking, LVEF, NT-proBNP, TC, LDL-C, HDL-C, eGFR, E/e’, and LVMi

*DM* diabetes mellitus, *SGLT-2i* sodium-glucose cotransporter-2 inhibitor, *BMI* body mass index, *CAD* coronary artery disease, *OSA* obstructive sleep apnea, *LVEF* left ventricular ejection fraction, *NT-proBNP* N-terminal pro-brain natriuretic peptide, *TC* total cholesterol, *LDL-C* low-density lipoprotein cholesterol, *HDL-C*, high-density lipoprotein cholesterol, *eGFR* estimated glomerular filtration rate, *E/e*’ the ratio of early diastolic mitral inflow velocity to septal mitral annulus tissue relaxation velocity in early diastole, *LVMi* left ventricular mass index

P values in bold are < 0.05

**Fig. 5:**
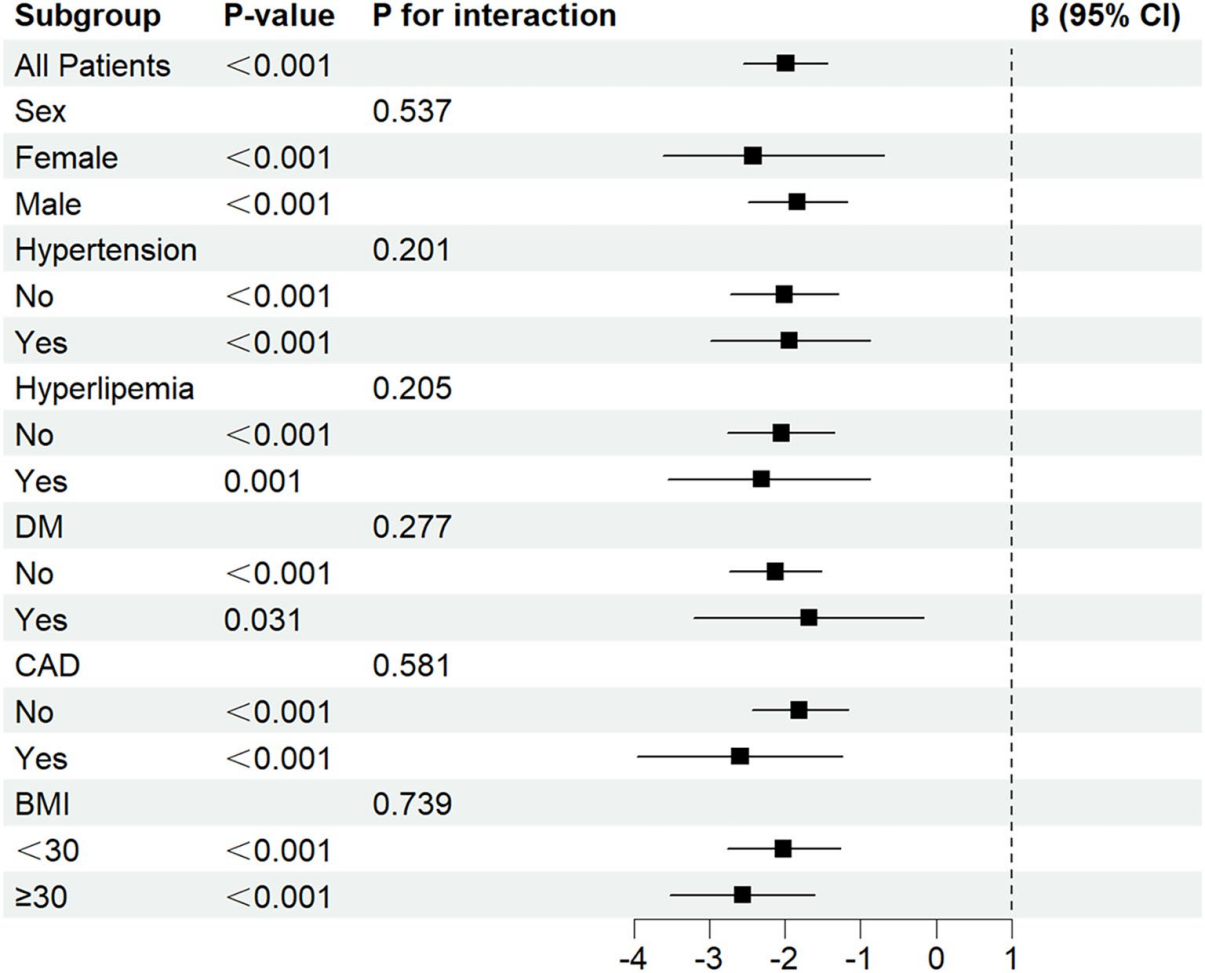
Subgroup analysis of the correlation between TyG index and GLS in patients with CHF. T*yG* index triglyceride-glucose index *GLS* global longitudinal strain, *CHF* chronic heart failure, *DM* diabetes mellitus, *CAD* coronary artery disease, *BMI* body mass index

The association between the TyG index and GLS for CHF was examined in subgroup analyses, and the calculated interaction P values are shown in Fig. 5. No significant interaction was found. Across various groups, the TyG index exhibited statistical significance in predicting reduced GLS, suggesting stability and consistency in the TyG index and GLS relationship. Additionally, subgroup analyses were conducted in HFrEF, HFmrEF, and HFpEF (Additional file 1: Table S7).

## Discussion

In the present study, we investigated the relationship between the TyG index and GLS in CHF, and further explored this association across different subgroups. After multivariable linear and logistic regression, the TyG index emerged as an independent indicator of reduced GLS in CHF. Elevated TyG index remained independently associated with reduced GLS in all three groups of patients with HFrEF, HFmrEF and HFpEF. This suggests that an elevated TyG index may be independently associated with more severe left ventricular insufficiency in CHF, regardless of specific classifications of CHF and the levels of LVEF.

A growing number of studies have suggested that GLS outperforms LVEF as a prognostic marker in HF patients [5, 11–13, 36]. A previous study demonstrated GLS’s super prognostic value for mortality compared to LVEF in patients with acute heart failure (AHF) [36]. GLS is associated with cardiac death and all-cause mortality in patients with CHF, independent of clinical features and cardiac structure and function [12]. Similarly, GLS has independently predicted poor long-term prognosis of HFrEF [11], and HFpEF [5].

Moreover, the Atherosclerosis Risk in the community study have highlighted the association between hemoglobin A1c (HbA1c) levels and GLS, indicating a nearly linear relationship [37]. A study analyzing the Framingham Heart Study found a significant association between IR and hypertriglyceridemia and GLS [38]. Previous study found that while DM itself may not be directly associated with GLS, elevated HbA1c levels have been independently associated with GLS, suggesting a potential direct impact of glucose metabolism on myocardial function [12]. Li et al. reported that IR is a critical component of metabolic syndrome (MetS), accounting for > 90% of the association between MetS and HF risk [39].

Previous studies have shown that IR is prevalent in patients with HF, and precedes the development of HF [40]. IR is also an indicator of HF and heart function deterioration [41]. IR contributes to adverse cardiac remodeling and dysfunction, thereby increasing cardiovascular risk by inducing glucose and lipid metabolism imbalances and triggering oxidative stress and inflammatory responses, endothelial dysfunction, and ectopic lipid accumulation [14, 15, 42–44]. The TyG index, serving as a reliable surrogate for IR, exhibit a robust association with homeostasis model assessment of HIEC [19–21].

Most previous studies investigating the impact of elevated TyG index on cardiac function have predominantly focused on non-HF patients and long-term adverse events in various populations. Recently, several cohort studies have confirmed that the TyG index can play a predictive role in the development of HF [25, 27, 45]. Chen et al. identified a significant association between higher TyG index and subclinical LV systolic dysfunction in T2DM patients with LVEF ≥ 50% [23]. Similarly, Huang et al. showed that elevated baseline and long-term TyG index levels were significantly linked to an increased risk of adverse LV remodeling, LV dysfunction and an increased risk of HF in a US community population without HF and CAD [22]. Additionally, a cross-sectional study confirmed an independent association between elevated TyG index and reduced GLS in patients with CAD, suggesting a potential association between the TyG index and subclinical left ventricular dysfunction in CAD patients [28].

SGLT-2 inhibitors could modify myocardial metabolism and have a favorable effect on LV function [46–48]. Recognizing the interplay between LV remodeling, diastolic function, and LVGLS in prediabetes and diabetes [38, 49]. we conducted a sensitivity analysis. This involved excluding patients using SGLT-2i and those with DM, which showed no change in the correlation between the TyG index and GLS. In different subgroups, elevated TyG index was statistically associated with significant reductions in GLS, suggesting stability and consistency of the relationship in patients with CHF.

The TyG index is an easy-to-measure parameter that predicts the incidence of HF in populations ranging from those without DM and CAD to those with DM and CAD [22, 26, 50] and has demonstrated diagnostic ability in distinguishing HFpEF patients from non-HFpEF individuals [51]. Meanwhile, previous studies have shown that the TyG index is associated with a poor long-term prognosis in HF [25, 27, 45]. The TyG index can therefore be considered as an additional tool in the routine clinical assessment of individuals at risk of HF. In resource-limited countries and settings, heart failure clinics could benefit from incorporating the TyG into their routine assessment. [52]. Given its ease of measurement, the TyG index can help in stratifying HF patient risk, ultimately enabling healthcare providers to provide more personalized care and tailored advice to patients.

This study revealed that higher TyG index levels were associated with an increased risk of left ventricular insufficiency in CHF and all three types of HF despite adjusting for confounding factors (e.g. age, sex, smoking, hypertension, and diabetes). We also found that the TyG index was associated with a reduced strain of HF in non-DM patients and among those excluding glucose- lowering and lipid-lowering medications, suggesting the stability of the relationship between the TyG index and reduced GLS. By using a novel IR alternative index to the TyG index, our study underscores the important role of IR in strain reduction in selected patients with HF, providing new insights into the pathogenesis of left ventricular insufficiency across the three types of HF.

## Limitation

Several limitations should be acknowledged in this study. (1) We acknowledged the limitations associated with the single-center, cross-sectional design of the study. This design may introduce bias, and we recognize the inability to assess whether the high TyG index group is more susceptible to subsequent adverse cardiovascular events compared to the low TyG index group. (2) While our study demonstrates relatively robust correlations between the TyG index and GLS in CHF across multiple multivariable models and subgroup analyses, we recognize the uncertainty regarding the utility of applying thees results to daily clinical practice. Further research is needed to determine the clinical implications of these findings. (3) We acknowledge the limitation posed by the different cutoff values for the TyG index among HF subgroups, which may restrict the generalization of the results to other populations. Understanding the variability of TyG index cutoffs across different HF subgroups is important for accurately interpreting and applying our findings in diverse clinical contexts. (4) We only analyzed patient GLS data and did not include assessments of left ventricular global circumferential strain or global radial strain, which could provide additional insights into cardiac function. (5) The use of a 2D scatter tracking technique for ultrasound image acquisition may be limited by acoustic window conditions, potentially impacting image quality and the analysis of hypoechoic regions. (6) Finally, we recognize the need for additional research to explore the relationship between the TyG index and GLS under the treatment of CHF. Investigating how the TyG index and GLS evolve in response to HF treatment could provide valuable insights into their clinical significance and potential therapeutic implications.

## Conclusion

In summary, our findings suggested that a high TyG index in CHF patients may be independently associated with clinically more pronounced LV dysfunction. Therefore, monitoring TyG index levels could be crucial to mitigate subsequent adverse outcomes.

### Supplementary Information

Below is the link to the electronic supplementary material.

Supplementary material 1. Figure S1 Comparison of GLS between high TyG index group and control group in three heart failure types groups. Figure S2 Scatterplot of GLS versus TyG index for three heart failure types. Figure S3 Restricted cubic spline plot between the TyG index level and reduced GLS for three heart failure types. Table S1 Co-linearity analysis between covariates. Table S2 Baseline characteristics of the study population according to the heart failure group. Table S3 Correlation between TyG index and cardiovascular risk factors in three heart failure types. Table S4 multivariable variable linear regression in three heart failure types. Table S5 Multivariable logistic regression analysis in three heart failure types. Table S6 Sensitivity analysis: exclusion of patient with DM, hypoglycemic or lipid-lowering drug use and SGLT-2i use in three heart failure types. Table S7 Subgroup analysis in three heart failure types.

## Data Availability

The datasets used and/or analyzed during the current study are available from the corresponding author on reasonable request.

## Abbreviations

GLS: Global longitudinal strain
LVEF: Left ventricular ejection fraction
LV: Left ventricular
HF: Heart failure
TyG index: Triglyceride-glucose index
IR: Insulin resistance
CHF: Chronic heart failure
FPG: Fasting plasma glucose
TG: Triglyceride
HFrEF: Heart failure with reduced ejection fraction
HFmrEF: Heart failure with mildly-reduced ejection fraction
HFpEF: Heart failure with preserved ejection fraction
HIEC: Hyperinsulinemic-euglycemic clamp
CAD: Coronary artery disease
LVESV: Left ventricular end systolic volume
LVEDV: Left ventricular end diastolic volume
LVDd: Left ventricular end diastolic dimension
LVDs: Left ventricular end systolic dimension
LVPWT: Left ventricular posterior wall thickness
IVST: Interventricular septal thickness
NT-proBNP: N-terminal pro-brain natriuretic peptide
SCr: Serum creatinine
BMI: Body mass index
DM: Diabetes mellitus
OGTT: Oral glucose tolerance test
CAG: Coronary angiography
OSA: Obstructive sleep apnea
CKD: Chronic kidney disease
eGFR: Estimated glomerular filtration rate
LVM: Left ventricular mass
BSA: Body surface area
LVMi: Left ventricular mass index
TC: Total cholesterol
LDL-C: Low-density lipoprotein cholesterol
HDL-C: High-density lipoprotein cholesterol
RCS: Restricted cubic spline
AHF: Acute heart failure
HbA1c: Hemoglobin A1c
