# Supplementary material for: Correlation between the triglyceride-glucose index and left ventricular global longitudinal strain in patients with chronic heart failure: a cross-sectional study
Source: Cardiovasc Diabetol. 2024 May 29;23:182. doi: 10.1186/s12933-024-02259-2 (PMC11137911; doi:10.1186/s12933-024-02259-2)
Supplement: Supplementary file 1 — Supplementary material 1. Figure S1 Comparison of GLS between high TyG index group and control group in three heart failure types groups. Figure S2 Scatterplot of GLS versus TyG index for three heart failure types. Figure S3 Restricted cubic spline plot between the TyG index level and reduced GLS for three heart failure types. Table S1 Co-linearity analysis between covariates. Table S2 Baseline characteristics of the study population according to the heart failure group. Table S3 Correlation between TyG index and cardiovascular risk factors in three heart failure types. Table S4 multivariable variable linear regression in three heart failure types. Table S5 Multivariable logistic regression analysis in three heart failure types. Table S6 Sensitivity analysis: exclusion of patient with DM, hypoglycemic or lipid-lowering drug use and SGLT-2i use in three heart failure types. Table S7 Subgroup analysis in three heart failure types. [file 12933_2024_2259_MOESM1_ESM.docx]

Figure S1 Comparison of GLS between high TyG index group and control group in three heart failure types groups


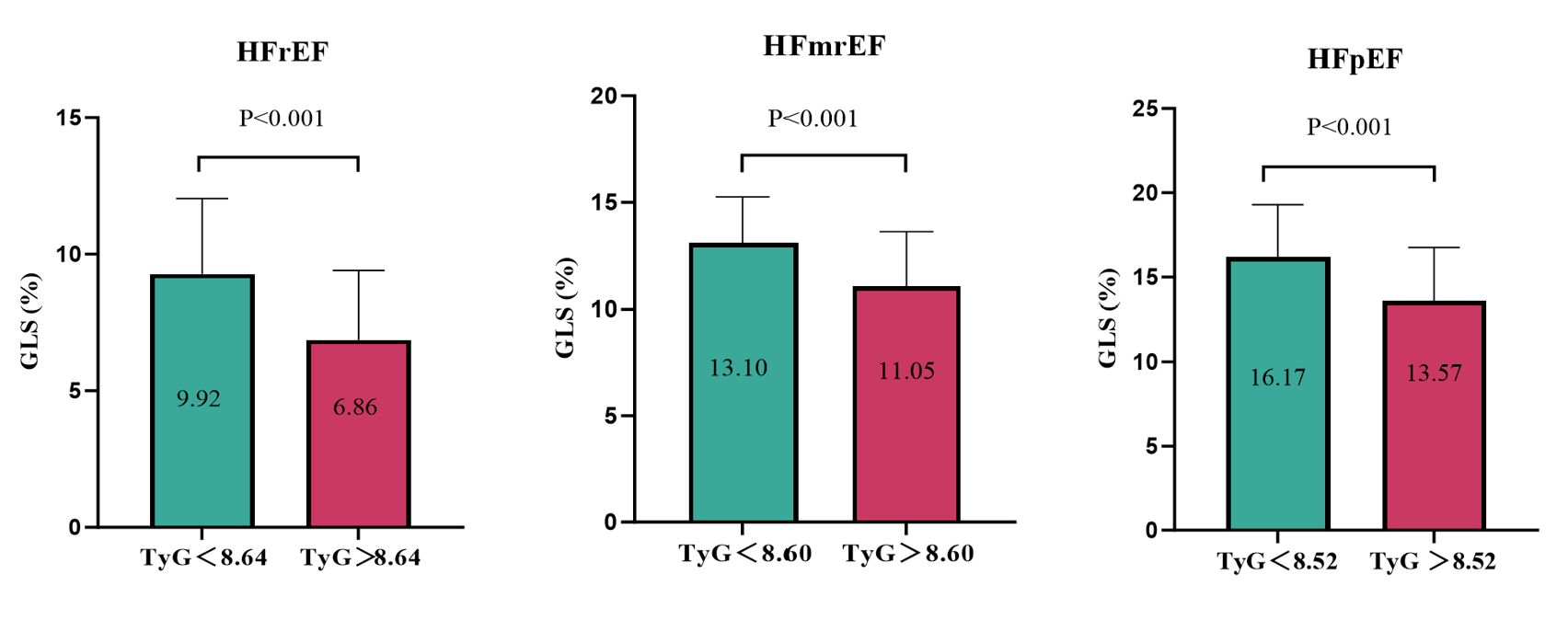


Figure S2 Scatterplot of GLS versus TyG index for three heart failure types


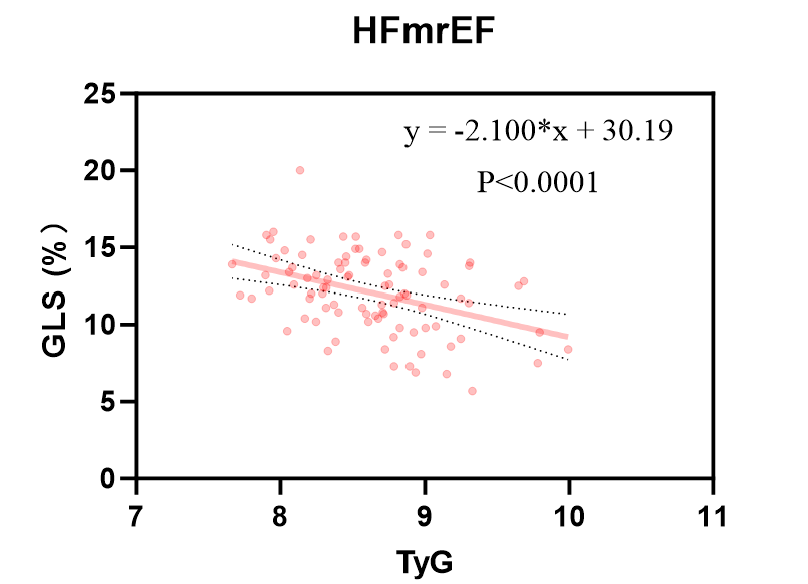

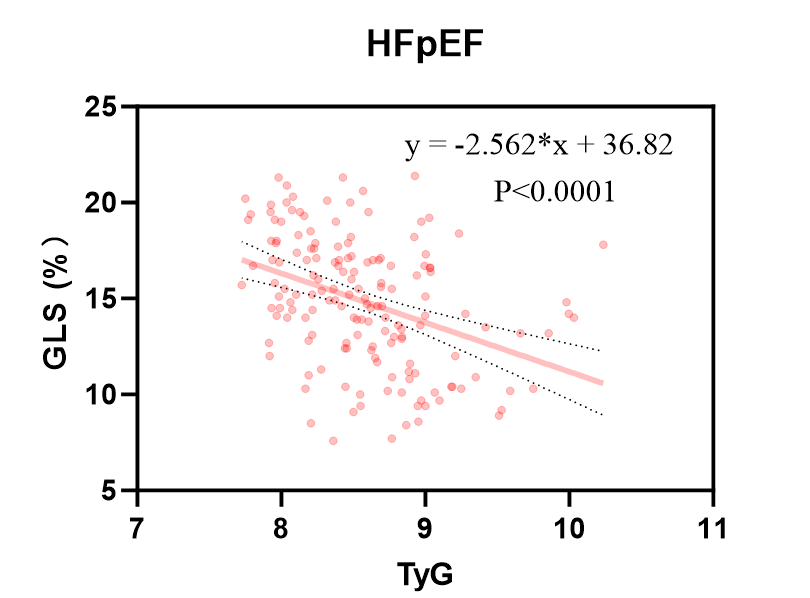

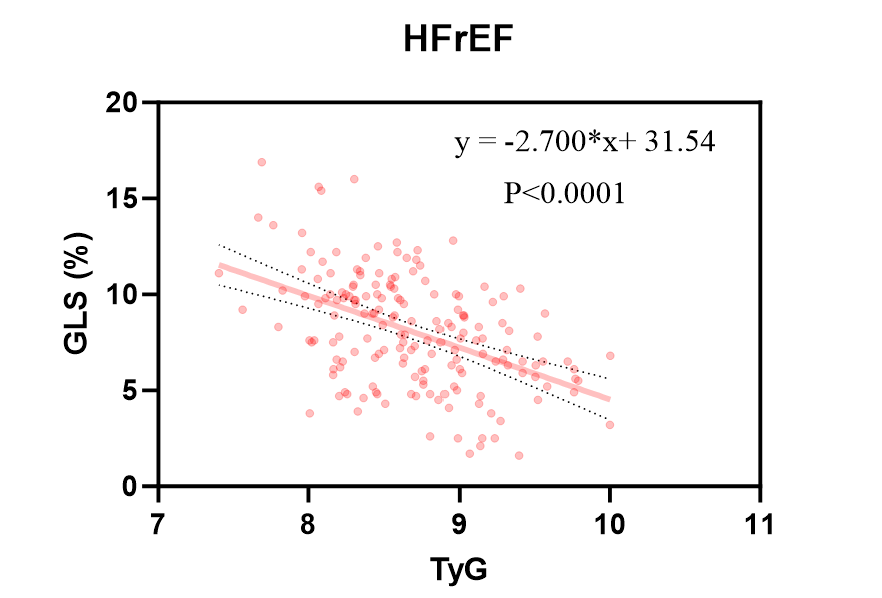


Figure S3 Restricted cubic spline plot between the TyG index level and reduced GLS for three heart failure types
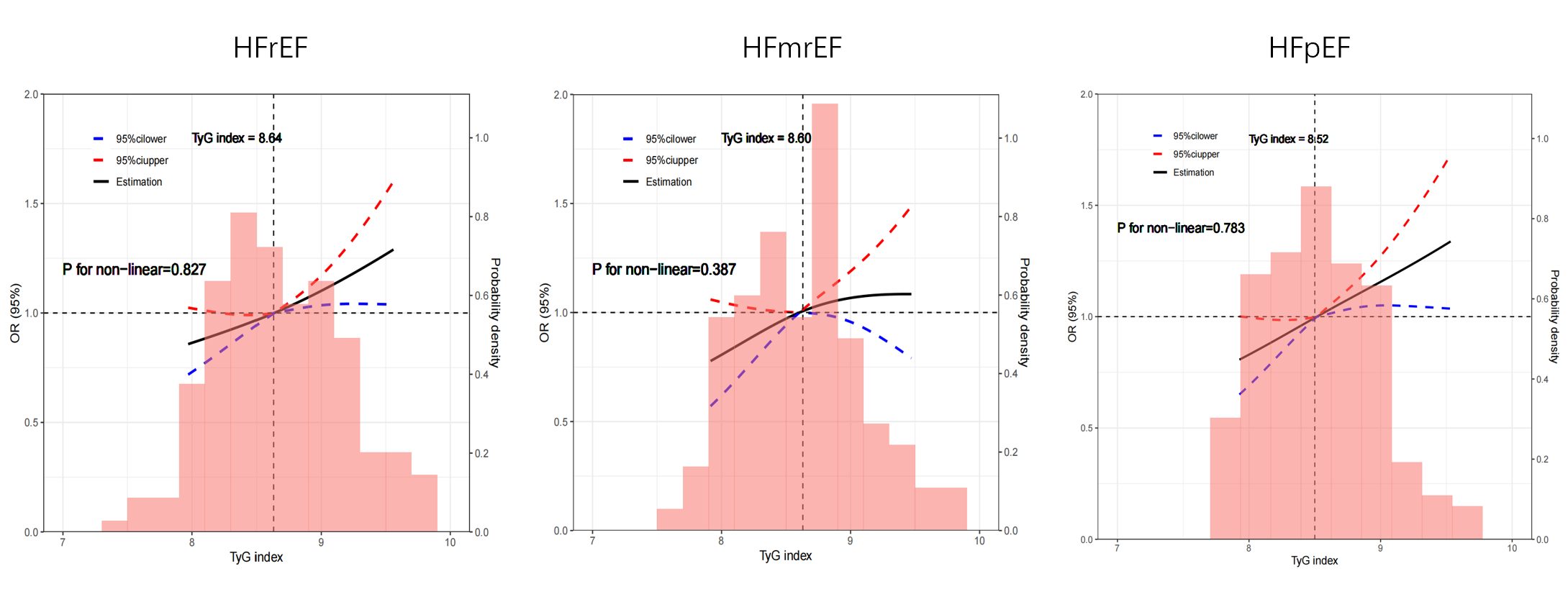


Adjusted for age, gender, BMI, hypertension, CAD, DM, hyperlipidemia, OSA, smoking, drinking, LVEF, NT-proBNP, TC, LDL-C, HDL-C, eGFR, E/e’, and LVMi

Reduced GLS in HFpEF: GLS＜7.9%；Reduced GLS in HFmrEF: GLS＜12.2%；Reduced GLS in HFpEF: GLS＜14.9%；

Table S1 Co-linearity analysis between covariates

| Covariates | Tolerance | VIF |
| --- | --- | --- |
| Age | 0.654 | 1.530 |
| Gender | 0.560 | 1.787 |
| BMI | 0.810 | 1.235 |
| Hypertension | 0.769 | 1.301 |
| DM | 0.821 | 1.219 |
| Hyperlipidemia | 0.816 | 1.225 |
| CAD | 0.742 | 1.348 |
| Smoking | 0.549 | 1.820 |
| Drinking | 0.535 | 1.870 |
| LVEF | 0.875 | 1.142 |
| TC | 0.495 | 2.019 |
| LDL-C | 0.492 | 2.031 |
| HDL-C | 0.944 | 1.059 |
| NT-proBNP | 0.815 | 1.227 |
| E/e’ | 0.797 | 1.255 |
| LVMi | 0.869 | 1.151 |

Table S2 Baseline characteristics of the study population according to the heart failure group

| Variables | HFrEF(N=175) | HFmrEF (N=92) | HFpEF (N=160) | P-value |
| --- | --- | --- | --- | --- |
| TyG index | 8.68±0.51 | 8.63±0.49 | 8.58±0.50 | 0.139 |
| General conditions |  |  |  |  |
| Age (years) | 48.23±13.82 | 47.54±14.58 | 49.00±15.00 | 0.736 |
| Male, n (%) | 119 (68.0) | 56 (60.9) | 96 (60.0) | 0.312 |
| BMI (kg/m^2^) | 26.92±6.36 | 26.66±6.30 | 28.06±6.46 | 0.116 |
| smoking, n (%) | 83 (47.7) | 40 (43.5) | 58 (38.3) | 0.231 |
| drinking, n (%) | 84 (48.3) | 45 (48.9) | 64 (41.6) | 0.387 |
| Medical history, n (%) |  |  |  |  |
| Hypertension | 62 (35.4) | 36 (39.1) | 58 (37.7) | 0.822 |
| DM | 27 (15.5) | 17 (18.5) | 24 (15.6) | 0.789 |
| Hyperlipidemia | 70 (40.0) | 32 (34.8) | 62 (38.8) | 0.702 |
| CAD | 28 (16.0) | 22 (23.9) | 39 (25.3) | 0.090 |
| Pervious PCI | 16 (9.1) | 12 (13.0) | 16 (10.4) | 0.612 |
| Pervious CABG | 1 (0.6) | 5 (5.4) | 7 (4.5) | **0.039** |
| OSA | 19 (10.9) | 9 (9.8) | 15 (9.7) | 0.935 |
| Echocardiographic |  |  |  |  |
| LVEF (%) | 31.00 (25.00-36.00) | 45.00 (44.00-47.00) | 56.00 (53.00-62.00) | **< 0.001** |
| GLS (%) | 8.08±2.92 | 12.08±2.57 | 14.87±3.40 | **< 0.001** |
| LVEDV (ml) | 190 (148-235) | 122 (104-155) | 111 (91-134) | **< 0.001** |
| LVSDV (ml) | 90 (57-130) | 65 (54-82) | 54 (44-68) | **< 0.001** |
| E (cm/s) | 77.00 (55.50-97.50) | 65.00 (52.00-78.00) | 73.00 (55.00-85.50) | **0.012** |
| E/e’ | 11.84 (9.60-16.13) | 8.20 (7.09-11.06) | 9.73 (8.77-13.35) | **< 0.001** |
| LVM (g) | 156.74 (91.76-264.69) | 176.33 (109.37-236.88) | 154.19 (106.46-207.09) | 0.521 |
| LVMi (g/m^2^) | 80.06 (48.81-145.48) | 89.20 (57.70-123.11) | 89.12 (59.67-124.12) | 0.605 |
| Laboratory text |  |  |  |  |
| FBG (mmol/L) | 5.40 (4.94-5.89) | 5.25 (4.90-5.89) | 5.51 (4.91-5.75) | 0.256 |
| TC (mmol/L) | 4.19 (3.48-4.97) | 3.88 (3.25-4.62) | 4.18 (3.45-4.98) | 0.066 |
| LDL-C (mmol/L) | 2.53±0.81 | 2.26±0.70 | 2.49±0.99 | 0.052 |
| HDL-C (mmol/L) | 1.08 (0.95-1.31) | 1.09 (0.91-1.49) | 1.13 (1.01-1.43) | **0.042** |
| TG (mmol/L) | 1.29 (1.01-1.78) | 1.30 (0.95-1.67) | 1.19 (0.89-1.51) | 0.086 |
| eGFR(ml/min/1.73m^2^) | 90.02±32.12 | 96.56±32.20 | 97.32±27.34 | 0.064 |
| NT-proBNP (pg/ml) | 1006 (422-2003) | 392 (102-928) | 261 (92-644) | **< 0.001** |
| Cardiovascular medications, n (%) |  |  |  |  |
| Hypoglycemic drugs | 26 (14.9) | 18 (19.6) | 24 (15.0) | 0.559 |
| Statins | 51 (29.1) | 23 (25.0) | 38 (23.8) | 0.510 |
| Beta-blockers | 161 (92.0) | 84 (91.3) | 119 (74.4) | **< 0.001** |
| ACEI/ARB/ANRI | 157 (89.7) | 69 (75.0) | 105 (65.6) | **< 0.001** |
| MRA | 151 (86.3) | 67 (75.0) | 88 (55.0) | **< 0.001** |
| SGLT-2i | 112 (64.0) | 43 (46.7) | 54 (33.8) | **< 0.001** |

P values in bold are＜0.05

Table S3 Correlation between TyG index and cardiovascular risk factors in three heart failure types

| Variables | Correlation coefficient (r) | P-value |
| --- | --- | --- |
| **HFrEF** |  |  |
| Age（years) | -0.191^&^ | **0.011** |
| BMI（kg/m^2^) | 0.233^&^ | **0.002** |
| TC (mmol/L) | 0.286* | **＜0.001** |
| LDL-C (mmol/L) | 0.298^&^ | **＜0.001** |
| HDL-C (mmol/L) | -0.299* | **0.003** |
| eGFR (ml/min/1.73m^2^) | 0.028^&^ | 0.710 |
| NT-proBNP (pg/ml) | -0.210* | **0.006** |
| LVEF (%) | -0.204* | **0.007** |
| GLS (%) | -0.471^&^ | **＜0.001** |
| **HFmrEF** |  |  |
| Age（years) | 0.092^&^ | 0.382 |
| BMI（kg/m^2^) | -0.090^&^ | 0.384 |
| TC (mmol/L) | -0.002* | 0.985 |
| LDL-C (mmol/L) | -0.048^&^ | 0.651 |
| HDL-C (mmol/L) | -0.163* | 0.133 |
| eGFR (ml/min/1.73m^2^) | -0.155^&^ | 0.140 |
| NT-proBNP (pg/ml) | -0.197* | 0.060 |
| LVEF (%) | -0.061* | 0.561 |
| GLS (%) | -0.398^&^ | **＜0.001** |
| **HFpEF** |  |  |
| Age（years) | 0.058 | 0.464 |
| BMI（kg/m^2^) | 0.014 | 0.864 |
| TC (mmol/L) | 0.267 | **0.001** |
| LDL-C (mmol/L) | 0.137 | 0.085 |
| HDL-C (mmol/L) | -0.310 | **＜0.001** |
| eGFR (ml/min/1.73m^2^) | -0.071 | 0.374 |
| NT-proBNP (pg/ml) | 0.085 | 0.297 |
| LVEF (%) | -0.033 | 0.679 |
| GLS (%) | -0.379 | **＜0.001** |

^&^Person

* Spearman

P values in bold are＜0.05

Table S4 multivariate linear regression in three heart failure types

|  | β | 95% CI | P-value |
| --- | --- | --- | --- |
| **HFrEF** |  |  |  |
| Model 1 | -2.65 | -3.43 to -1.86 | < 0.001 |
| Model 2 | -2.75 | -3.57 to -1.94 | < 0.001 |
| Model 3 | -2.22 | -2.99 to -1.44 | < 0.001 |
| **HFmrEF** |  |  |  |
| Model 1 | -2.00 | -3.01 to -1.01 | < 0.001 |
| Model 2 | -2.00 | -3.12 to -0.89 | 0.001 |
| Model 3 | -2.10 | -3.27 to -0.93 | 0.001 |
| **HFpEF** |  |  |  |
| Model 1 | -2.35 | -3.32 to –1.38 | < 0.001 |
| Model 2 | -2.34 | -3.39 to -1.29 | < 0.001 |
| Model 3 | -2.46 | -3.58 to -1.35 | < 0.001 |

Model 1: adjusted for age and gender

Model 2: adjusted for variables :model 1 covariates + BMI, hypertension, CAD, DM, hyperlipidemia, OSA, smoking, drinking

Model 3: adjusted for variables :model 2 covariates+ LVEF, NT-proBNP, TC, LDL-C, HDL-C, eGFR, E/e’, and LVMi

P values in bold are＜0.05

Table S5 Multivariate logistic regression analysis in three heart failure types

| TyG | OR (95% CI) |  |  |
| --- | --- | --- | --- |
|  | Model 1 | Model 2 | Model 3 |
| HFrEF |  |  |  |
| Per 1 Unit increase | 5.08 (2.44-10.56)*** | 6.00 (2.69-13.39) *** | 5.30 (1.86-15.10) ** |
| Tertile 1 | Ref. | Ref. | Ref. |
| Tertile 2 | 2.03 (0.94-4.39) | 1.95 (0.87-4.38) | 1.81 (0.62-5.31) |
| Tertile 3 | 4.35 (1.94-9.76) *** | 4.74 (2.01-11.17)*** | 4.27 (1.34-13.62)* |
| P for trend | **0.002** | **0.002** | **0.045** |
| HFmrEF |  |  |  |
| Per 1 Unit increase | 2.61 (1.03-6.64) | 3.29 (0.93-5.26) | 3.51 (0.86-8.38) |
| Tertile 1 | Ref. | Ref. | Ref. |
| Tertile 2 | 1.38 (0.50-3.82) | 1.82 (0.54-4.06) | 3.52 (0.72-6.78) |
| Tertile 3 | 2.57 (0.87-7.59) | 5.01 (1.19-9.56)** | 8.57 1.50-15.67)* |
| P for trend | 0.224 | 0.086 | 0.054 |
| HFpEF |  |  |  |
| Per 1 Unit increase | 5.16 (2.33-11.45)*** | 5.99 (2.40-14.97) *** | 6.36 (2.12-19.14) ** |
| Tertile 1 | Ref. | Ref. | Ref. |
| Tertile 2 | 2.05 (0.90-4.65) | 1.64 (0.68-3.97) | 2.21 (0.75-6.47) |
| Tertile 3 | 5.50 (2.31-13.13) *** | 6.69 (2.42-18.47)*** | 7.13 (2.03-25.19)** |
| P for trend | **0.001** | **0.001** | **0.009** |

Model 1: adjusted for age and gender

Model 2: adjusted for variables :model 1 covariates + BMI, hypertension, CAD, DM, hyperlipidemia, OSA, smoking, drinking

Model 3: adjusted for variables :model 2 covariates+ LVEF, NT-proBNP, TC, LDL-C, HDL-C, eGFR, E/e’, and LVMi

*P＜0.05

**P＜0.01

***P＜0.001

P values in bold are＜0.05

Table S6 Sensitivity analysis: exclusion of patient with DM, hypoglycemic or lipid-lowering drug use and SGLT-2i use in three heart failure types

|  | β | 95% CI | P-value |
| --- | --- | --- | --- |
| Group 1 |  |  |  |
| HFrEF | -2.65 | -3.43 to -1.87 | ＜0.001 |
| HFmrEF | -1.84 | -3.23 to -0.45 | 0.010 |
| HFpEF | -2.36 | -3.60 to -1.11 | ＜0.001 |
| Group 2 |  |  |  |
| HFrEF | -2.64 | -3.64 to –1.64 | ＜0.001 |
| HFmrEF | -2.02 | -3.61 to -0.43 | 0.014 |
| HFpEF | -1.72 | -3.15 to -0.29 | 0.019 |
| Group 3 |  |  |  |
| HFrEF | -1.75 | -3.11 to -0.38 | 0.014 |
| HFmrEF | -2.43 | -4.69 to -0.17 | 0.036 |
| HFpEF | -2.53 | -4.06 to -0.99 | 0.002 |

Adjusted for age, gender, BMI, hypertension, CAD, hyperlipidemia, OSA, smoking, drinking, LVEF, NT-proBNP, TC, LDL-C, HDL-C, eGFR, E/e’, and LVMi

Group 1:patients without DM

Group 2: patients without hypoglycemic or lipid-lowering drug use

Group 3: patients without SGLT-2i use

P values in bold are < 0.05

Table S7 Subgroup analysis in three heart failure types

|  | HFrEF |  | HFmrEF |  | | HFpEF |  | |
| --- | --- | --- | --- | --- | --- | --- | --- | --- |
|  | β (95% CI) | P for interaction | β (95% CI) | | P for interaction | β (95% CI) | | P for interaction |
| Gender |  | 0.781 |  | | 0.994 |  | | 0.083 |
| Female | -2.68 (-4.24 to -1.12)** |  | -2.34 (-5.81 to 1.12) | |  | -2.89 (-4.48 to -1.30)** | |  |
| Male | -2.82 (-3.84 to -1.81)*** |  | -2.08 (-3.34 to -0.81)** | |  | -1.81 (-3.14 to -0.49)** | |  |
| DM |  | 0.222 |  | | 0.149 |  | | 0.487 |
| No | -2.70 (-3.69 to -1.71)*** |  | -1.82 (-3.05 to -0.60)** | |  | -2.08 (-3.24 to -0.93)** | |  |
| Yes | -2.33 (-4.40 to -0.68)** |  | -4.40 (-8.49 to 0.64) | |  | -3.38 (-7.34 to 0.58) | |  |
| HTN |  | 0.834 |  | | 0.917 |  | | 0.963 |
| No | -2.32 (-3.19 to -1.47)*** |  | -1.82 (-3.41 to -0.21)* | |  | -2.41 (-3.78 to -1.04)** | |  |
| Yes | -0.83 (-2.53 to -0.87) |  | -1.69 (-3.79 to 0.42) | |  | -2.41 (-4.28 to -0.55)* | |  |
| Hyperlipidaemia |  | 0.429 |  | | 0.867 |  | | 0.122 |
| No | -3.17 (-4.30 to -2.01)*** |  | -2.31 (-3.81 to -0.82)* | |  | -2.58 (-3.77 to -1.38 )*** | |  |
| Yes | -2.20 (-3.42 to -0.97)** |  | -1.48 (-3.43 to -0.47) | |  | -1.58 (-3.79 to 0.64) | |  |
| CAD |  | 0.306 |  | | 0.871 |  | | 0.661 |
| No | -2.52 (-3.43 to -1.64)*** |  | -2.03 (-3.28 to -0.78)* | | 0 | -2.42 (-3.70 to -1.13)*** | |  |
| Yes | -3.93 (-6.63 to -1.22) |  | -3.82 (-7.83 to 0.19) | |  | -1.49 (-3.56 to 0.59) | |  |
| OSA |  | **0.046** |  | | 0.748 |  | | 0.714 |
| No | -3.00 (-3.83 to -2.71)*** |  | -2.02 (-3.28 to -0.75)* | |  | -2.40 (-3.49 to -1.31)*** | |  |
| Yes | 1.34 (-2.73 to 4.10) |  | -3.18 (-5.12 to 1.34) | |  | -1.64 (-9.20 to 5.92) | |  |
| BMI |  | 0.293 |  | | 0.134 |  | | 0.363 |
| ＜30 | -2.74 (-1.55 to -1.81)*** |  | -2.11 (-3.40 to -0.81)* | |  | -2.28 (-3.47 to -1.08)*** | |  |
| ≥30 | -1.19 (-7.56 to 2.54) |  | -1.06 (-4.09 to 1.96) | |  | -2.66 (-8.00 to 2.68) | |  |

Adjusted for age, gender, BMI, HTN,CAD, DM, hyperlipidemia, OSA, smoking, and drinking

* P < 0.05

** P < 0.01

*** P < 0.001

P values in bold are < 0.05
